# Virulence Plasmids of *Rhodococcus equi* Isolates From Cuban Patients With AIDS

**DOI:** 10.3389/fvets.2021.628239

**Published:** 2021-02-25

**Authors:** Daniel Salazar-Rodríguez, Yamilé Aleaga-Santiesteban, Enrique Iglesias, Arturo Plascencia-Hernández, Héctor R. Pérez-Gómez, Enrique J. Calderón, José A. Vázquez-Boland, Yaxsier de Armas

**Affiliations:** ^1^Department of Clinical Microbiology Diagnostic, Hospital Center of Institute of Tropical Medicine “Pedro Kourí,” Havana, Cuba; ^2^Departamento de Vacunas, Centro de Ingeniería Genética y Biotecnología, Havana, Cuba; ^3^Centro Universitario de Ciencias de la Salud de la Universidad de Guadalajara, Guadalajara, Mexico; ^4^Instituto de Biomedicina de Sevilla, Hospital Universitario Virgen del Rocío/Consejo Superior de Investigaciones Científicas/Universidad de Sevilla, Centro de Investigación Biomédica en Red de Epidemiología y Salud Pública (CIBERESP), Seville, Spain; ^5^Microbial Pathogenesis Group, Edinburgh Medical School (Biomedical Sciences - Infection Medicine), University of Edinburgh, Edinburgh, United Kingdom; ^6^Pathology Department, Hospital Center of Institute of Tropical Medicine “Pedro Kourí,” Havana, Cuba

**Keywords:** *Rhodococcus equi*, aids, human immunodeficiency virus, Cuba, animal host-associated virulence plasmids, TRAVAP virulence plasmid typing, *R. equi* zoonotic infection

## Abstract

*Rhodococcus equi* is an animal pathogen and zoonotic human opportunistic pathogen associated with immunosuppressive conditions. The pathogenicity of *R. equi* is linked to three animal host-associated virulence plasmids encoding a family of “Virulence Associated Proteins” (VAPs). Here, the PCR-based TRAVAP molecular typing system for the *R. equi* virulence plasmids was applied to 26 *R. equi* strains isolated between 2010 and 2016 at the Institute of Tropical Medicine “Pedro Kourí,” Cuba, from individuals living with HIV/AIDS. TRAVAP detects 4 gene markers, *traA* common to the three virulence plasmids, and *vapA, vapB*, and *vapN* specific to each of the host-associated plasmid types (equine pVAPA, porcine pVAPB, and ruminant pVAPN). Of the 26 isolates, six were positive to the *vapB* (porcine-type) marker, 4 (15.4%) to the *vapA* (equine-type) marker, and 1 (3.8%) to the *vapN* (ruminant-type) marker. Most of the isolates 14 (53.8%) were negative to all TRAVAP markers, suggesting they lacked a virulence plasmid. To our knowledge, this work is the first to report the molecular characterization of *R. equi* isolates from Cuba. Our findings provide insight into the zoonotic origin of *R. equi* infections in people and the potential dispensability of the virulence plasmid in immunosuppressed patients.

## Introduction

*Rhodococcus equi* is a well-known horse pathogen first described in 1923 by Magnusson as the causative agent of pyogranulomatous pneumonia and lung abscesses in foals aged <6 months (1). *R. equi* also infects other mammals including pigs, cattle, goats, cats, dogs, and humans, in which the infection is believed to be zoonotic (2). The first case of human infection was reported in an adult receiving immunosuppressive therapy in 1966. In the 1980–90's, *R. equi* emerged as an opportunistic pathogen associated with AIDS and other immunosuppresive conditions such as organ transplant chemotherapy and steroid therapy. In humans, *R. equi* causes purulent bronchopneumonia and occasional extrapulmonary pyogenic infections (3, 4).

*R. equi* is widelspread in nature, inhabits soil and colonizes the intestine of grazing animals and omnivores. The most likely transmission mechanism is inhalation of the bacteria in aerosolized dust particles. In addition, the accidental inoculation of the microorganism in injuries, mucous membranes or ingestion of contaminated food are other possible routes of infection (5). The pathogen is a facultative intracellular parasite capable of replicating within macrophages, thus evading host defense mechanisms (2, 6, 7).

The pathogenicity of *R. equi* is associated with the presence of a virulence plasmid encoding a family of “Virulence Associated Proteins” (VAPs) (2, 6–8). Two different *R. equi* circular virulence plasmid variants were initially characterized: the VapA-encoding pVAPA, found in virulent isolates of equine origin, and the VapB-encoding pVAPB, found in isolates showing intermediate virulence in mice, recovered from submaxillary lymph nodes of slaughtered pigs and human clinical specimens (2, 9–11). Recently, the VapN-encoding linear virulence plasmid, pVAPN, was identified in isolates from bovids and human clinical specimens (12). These *R. equi* virulence plasmids are animal host-specific, with pVAPA being associated with horses, pVAPB with pigs and pVAPN with cattle (ruminants) (2, 8, 11–13). Human isolates, in contrast, can carry any of the three animal host-associated plasmid types. This finding suggested that humans were accidental hosts for *R. equi* and that human infections had a zoonotic origin (2, 8, 13).

Molecular typing of *R. equi* is insufficiently developed and little is known about the epidemiology and transmission of this multihost pathogen (13). Thus far, there has only been one case report of a human *R. equi* infection in Cuba (14). In this study, we used the PCR-based virulence plasmid typing system developed by Ocampo-Sosa et al. (13), complemented with the novel vapN marker for the ruminant-associated virulence plasmid pVAPN (12), to analyze the virulence plasmid carriage of a series of human *R. equi* strains recently isolated from people living with HIV in Cuba.

## Materials and Methods

A case series study of 26 isolates of *R. equi* from 26 people living with HIV/AIDS was conducted during the period between January 2010 and December 2016. These isolates were obtained from all the patients with *R. equi* / HIV coinfection (one isolate/patient) admitted to the Hospital Center of Institute of Tropical Medicine “Pedro Kourí” (IPK) during this 7-year period. The isolates belong to the collection of the Department of Clinical Microbiology Diagnostic of the Hospital Center of IPK, Havana, Cuba. No data were available in the clinical records of previous contact of patients with farm animals or farm environments.

*R. equi* isolates were stored at −70°C and revived by incubation in Muller-Hilton broth at 37°C for 24 h. The colonies from the isolates were used for DNA purification using QIAmp kit (Qiagene, Hilden, Germany) following manufacturer's instructions.

The DNA extracted from each isolate (50 ng) was PCR-amplified in a reaction volume of 50 μL, using the *traA*-, *vapA*-, and *vapB*-specific primers of the TRAVAP typing system (13) updated with additional primers for the *vapN* gene marker for the novel pVAPN plasmid (12). The sequences of the primers are shown in Supplementary Table 1, PCR reactions were performed using the modifications reported elsewhere (15). Previously, the isolates were confirmed as *R. equi* based on the amplification of a fragment of the *choE* gene encoding cholesterol oxidase (16).

For the detection of each PCR product, 15 μL of the resulting mixture was run on a 1.6% agarose gel containing ethidium bromide. The visualization of the amplicons was carried out by exposing the gel to ultraviolet light in a transilluminator equipment (Macrovue 2011, LKB, Sweden).

The study was approved by the Ethic Committee of Institute of Tropical Medicine “Pedro Kourí” (CEI-IPK 51-18). It was conducted in accordance with national regulations and the Declaration of Helsinki. All participants signed a written informed consent.

## Results

The analysis of the 26 isolates of *R. equi* showed that 4 (15.4%) were positive for the *vapA* gene of the equine-associated pVAPA virulence plasmid. The porcine-associated pVAPB plasmid was identified in six isolates (23.1%), as determined by a positive reaction for the *vapB* gene marker. One isolate gave a positive PCR with the *vapN* marker, suggesting it carried the ruminant-associated pVAPN plasmid. The *traA* gene was detected in a total 11 of the 26 cases analyzed (42.3%). Most isolates in this investigation (53.8%) were TRAVAP negative (i.e., negative to the *traA, vapA, vapB*, and *vapN* PCR markers). The classification of the isolates according to the TRAVAP system is shown in Supplementary Table 2 and the percentage distribution of each virulence plasmid type in the analyzed sample in Supplementary Table 3.

## Discussion

In this study, we provide the first molecular characterization of *R. equi* isolates in people living with HIV/AIDS in Cuba, with a focus on the molecular analysis of the animal host-adapted virulence plasmids using the PCR-based TRAVAP typing method (13). These plasmids, designated pVAPA, pVAPB, and pVAPN (2, 11, 12), enable *R. equi* to, respectively, colonize horses, pigs, and ruminants, while they can at the same time be indistinctly found in isolates recovered from non-adapted animal species (i.e., the case of humans) (2, 8). Because these plasmids are associated with specific animal hosts, it is in principle possible to infer the source of zoonotic transmission of human *R. equi* isolates by determining the type of virulence plasmid they carry. The molecular typing of the *R. equi* virulence plasmids has therefore recently emerged as a tool of great epidemiological importance that can provide valuable insight into the possible sources of human rhodococcal infections (2, 12, 13).

A previous survey involving a global collection of *R. equi* isolates showed that about half of human strains carried a pVAPB (porcine type) plasmid (13), suggesting that exposure to pig farms is a major risk factor. This is consistent with our finding that most of the virulence plasmid-positive Cuban isolates carred the pVAPB plasmid type. Our results are comparable to those of previous studies by Takai et al. in Thailand (10), and Ribeiro et al. in Brazil (17, 18), also involving human isolates from HIV-infected patients, which found that the most abundant plasmid type in these isolates was the porcine (*vapB*^+^) plasmid type, pVAPB. This indicates that the infection by *R. equi* in the sample of Cuban people living with HIV/AIDS involves, like in other countries, the pig as the probable main source of transmission of the microorganism. Pork is one of the main sources of protein intake by Cuban people and many individuals are in close contact with pigs for breeding and meat trade.

The first of the Vap antigen-encoding virulence plasmids to be identified, by Takai et al. in 1991, was the equine-associated pVAPA (9, 19). This plasmid is as an essential virulence determinant of the pathogen in foals and mice (20). In the present work, four human isolates carrying the pVAPA plasmid were identified. This fact suggests that contact with horses might be another significant source of *R. equi* infection in humans in Cuba (transportation by horse-drawn vehicles is a common practice in rural areas), although of comparatively lesser importance than pigs or pig farm environments.

The *traA* gene, used in the TRAVAP typing scheme as an indicator of “presence of a virulence plasmid” (13), encodes a putative conjugative relaxase that is conserved in the three host-associated virulence plasmid types (11, 12). Ocampo et al. demonstrated that of 89 *vapA/B*-negative strains, 40 (44.9%) tested positive for the *traA* gene, indicating that they could also contain a plasmid (13). This *traA*^+^/*vapAB*^−^ genotype was later shown to correspond to strains carrying the bovine (ruminant)-associated pVAPN (12). In the study by Ocampo-Sosa et al., as many as 26% of the human isolates analyzed carried the bovine-associated *traA*^+^/*vapAB*^−^ (pVAPN) plasmid, suggesting that cattle farm environments are a significant source of human *R. equi* infections. This does not seem to be the case in Cuba, because in the series of 26 isolates analyzed here, only one (3.8%) was positive to the *vapN* marker. Our data are comparable to those of Ribeiro et al. in Brazil, who found that only two out of 74 *R. equi* strains isolated from the lungs of HIV/AIDS patients in the period 1997–2016 carried the *vapN*^+^ (pVAPN) plasmid (17).

It must be noted that before the introduction of the *traA* marker in 2006 (13), many of the *traA*^+^/*vapAB*^−^ (pVAPN) strains—mostly bovine and human clinical isolates—were initially considered to be devoid of a virulence plasmid. This means that all the *R. equi* literature prior to the discovery of the *traA* (13) and *vapN* (12) gene markers, particularly those studies reporting “avirulent/plasmidless” human isolates, must be interpreted with caution. Nevertheless, a significant proportion of *R. equi* human isolates (23 to 43%) actually appear to be devoid of a virulence plasmid, as judged by their negative reaction to the TRAVAP markers (13, 15). This seems to be the case for most of the human strains analyzed in our study, in which the TRAVAP-negative genotype clearly predominated (53.8% of isolates). This high percentage of “avirulent/plasmidless” isolates could be explained by the increased susceptibility of people living with HIV, which may render the virulence plasmid dispensable for *R. equi* to cause an infection. About half of environmental soil isolates of *R. equi* lack a virulence plasmid (13), presumably because it imposes a fitness cost during saprophytic growth in the absence of host selection (2, 8). It is therefore possible to speculate that the *R. equi* strains found in human patients could primarily be non-virulent environmental isolates not directly associated with an animal host. Alternatively, the strains could have lost the plasmid during subculturing in the laboratory. Indeed, spontaneous virulence plasmid curing can be observed during *in vitro* growth of *R. equi* at 37°C (21, 22). However, it cannot be excluded that some of the 14 TRAVAP-negative isolates might harbor some unknown plasmid encoding novel virulent determinants.

Finally, one isolate was positive to *traA* but negative to each of the plasmid type-specific markers *vapA, vapB*, and *vapN*. A similar situation was previously observed in a human isolate in a study performed in the United State of America (15), suggesting that there might be microvariability in some of the virulence plasmids' PCR target sequences. An alternative and more interesting possibility is that this may reflect the existence of an additional virulence plasmid type(s) yet to be characterized. It is also worth noting that the *vapN*^+^ isolate was negative for *traA*, suggesting the existence of microvariability in the *traA* sequence (the *traA* gene is actually a pseudogene in the pVAPN plasmid and is thus theoretically prone to genetic drift) (12). The possibility of TRAVAP gene marker variability may also in part account for the lack of detection of the virulence plasmid in a number of the “avirulent/plasmidless” *R. equi* isolates.

Our study has three limitations: (i) small sample size (*n* = 26), although the isolates were obtained in a period of 7 years and included all cases of *R equi* / HIV coinfection admitted at IPK hospital; (ii) we did not isolate/sequence the virulence plasmids, which would have provided insight into their genetic makeup or the variability underlying the unusual *traA*^+^/*vapABN*^−^ and *traA*^−^/v*apAB*^−^/*vapN*^+^ TRAVAP genotypes; and (iii) a history of contact with farm animals, manure or occupational exposure to farm environments was not available from the clinical records. In any case, this study has value in that it is the first, to our knowledge, in reporting the molecular characterization of *R. equi* isolates in people living with HIV/AIDS in Cuba, and indeed in the Caribbean islands.

In summary, our study provides interesting insight into possible animal sources of *R. equi* infection in AIDS patients from Cuba, of value to public health authorities and, more generally, in the interpretation of the epidemiology of *R. equi* infections in people.

## Data Availability Statement

The raw data supporting the conclusions of this article will be made available by the authors, without undue reservation.

## Ethics Statement

The studies involving human participants were reviewed and approved by Comité de Ética del Instituto Pedro Kouri. The patients/participants provided their written informed consent to participate in this study.

## Author Contributions

YA, EC, and JV-B: conceptualization and supervision. DS-R, YA-S, EI, JV-B, and YA: methodology. AP-H and HP-G: formal analysis. YA, EI, EC, and JV-B: data curation and writing—review and editing. DS-R, YA-S, AP-H, and HP-G: writing—original draft preparation. DS-R and YA-S: visualization. All authors have read and agreed to the published version of the manuscript.

## Conflict of Interest

The authors declare that the research was conducted in the absence of any commercial or financial relationships that could be construed as a potential conflict of interest.
