# Supplementary material for: Virulence Plasmids of Rhodococcus equi Isolates From Cuban Patients With AIDS
Source: Front Vet Sci. 2021 Feb 25;8:628239. doi: 10.3389/fvets.2021.628239 (PMC7947234; doi:10.3389/fvets.2021.628239)
Supplement: Supplementary file 1 [file Data_Sheet_1.pdf]

Supplementary Material

1 Supplementary Figures and Tables

Table 1. Oligonucleotide primers used for *R. equi* virulence plasmid TRAVAP typing scheme (13) with indication of the PCR product size for each target gene. Nucleotide sequences were derived from genomic information of the virulence plasmids (9,11,12). The *traA* primers were as described in Ocampo-Sosa et al. (13), *vapA*/*B*/*N* primers as modified in (15).

Gene	Sequence primers (5'-3')	Size PCR amplicon (bp)
vapA	F-AGACTCTTCACAAGACGGTTTCT<br>R-TCGCCATCGAAGACCTTTCCTT	334
vapB	F-CTTCTTAAGGATGGGGCAGG<br>R-GGCTACCTTCAGCCTGCTAT	485
vapN	F-GGTACTGCAGGCAACTGCTA<br>R-GAGCTGCTACTACCGTGGTC	425
traA	F-AGAGTTCATGCGTGACAACG<br>R-GTCCACAGGTCACCGTTCTT	959

Table 2. Virulence plasmid genotype for each of the *R. equi* isolates analyzed according to the TRAVAP plasmid typing system.

Isolate	TRAVAP genotype	Isolate	TRAVAP genotype
1	$traA^{+}/vapAN^{-}/vapB^{+}$	14	$traA^{+}/vapAN^{-}/vapB^{+}$
2	$traA^{-}/vapABN^{-}$	15	$traA^{-}/vapABN^{-}$
3	$traA^{+}/vapABN^{-}$	16	$traA^{-}/vapABN^{-}$
4	$traA^{+}/vapAN^{-}/vapB^{+}$	17	$traA^{-}/vapABN^{-}$
5	$traA^{-}/vapABN^{-}$	18	$traA^{-}/vapABN^{-}$
6	$traA^{-}/vapABN^{-}$	19	$traA^{+}/vapAN^{-}/vapB^{+}$
7	$traA^{-}/vapABN^{-}$	20	$traA^{-}/vapABN^{-}$
8	$traA^{+}/vapA^{+}/vapBN^{-}$	21	$traA^{-}/vapAB^{-}/VapN^{+}$
9	$traA^{-}/vapABN^{-}$	22	$traA^{+}/vapAN^{-}/vapB^{+}$
10	$traA^{+}/vapA^{+}/vapBN^{-}$	23	$traA^{+}/vapAN^{-}vapB^{+}$
11	$traA^{+}/vapA^{+}/vapBN^{-}$	24	$traA^{-}/vapABN^{-}$
12	$traA^{-}/vapABN^{-}$	25	$traA^{-}/vapABN^{-}$
13	$traA^{+}/vapA^{+}/vapBN^{-}$	26	$traA^{-}/vapABN^{-}$

Table 3. Summary of virulence plasmid types identified in the Cuban isolates based on the TRAVAP plasmid typing results.

Pattern	TRAVAP genotype	Virulence plasmid	n (%)
I	*traA*$^{-}$/*vapABN*$^{-}$	no plasmid	14 (53.8%)
II	*traA*$^{+}$/*vapAN*$^{-}$/*vapB*$^{+}$	**pVAPB** - porcine type	6 (23.1%)
III	*traA*$^{+}$/*vapA*$^{+}$/*vapBN*$^{-}$	**pVAPA** - equine type	4 (15.4%)
IV	*traA*$^{-}$/*vapAB*$^{-}$/*vapN*$^{+}$	**pVAPN** - ruminant type (with *traA*$^{-}$ marker)	1 (3.8%)
V	*traA*$^{+}$/*vapABN*$^{-}$	?	1 (3.8%)
